# One-Step Labeling Based on Eu-MOFs to Develop Fluorescence Side-Flow Immunoassay for AFB1 Detection in Corn

**DOI:** 10.3390/bios15050313

**Published:** 2025-05-14

**Authors:** Yinjun Li, Hua Ding, Ziyu Wang, Zewei Luo, Xitian Peng

**Affiliations:** 1 Institute of Agricultural Quality Standards and Testing Technology Research, Hubei Academy of Agricultural Sciences, Hubei Key Laboratory of Nutritional Quality and Safety of Agro Products, Wuhan 430064, China; yinjunli2015@163.com (Y.L.);; 2 Research Center of Analytical Instrumentation, School of Mechanical Engineering, Sichuan University, Chengdu 610065, China

**Keywords:** lateral flow immunoassay, metal–organic frameworks, aflatoxin B1, rapid detection

## Abstract

Lateral flow immunoassay (LFIA) is a promising tool for rapid detection in the field of agricultural product analysis due to its advantages of cost-effectiveness and operational simplicity. In this work, Eu metal–organic frameworks (MOFs) were introduced to LFIA as a rapid detection method characterized by high stability and low interference. Key research objectives included strong fluorescence, ease of labeling, and the utilization of fluorescent probes. Eu-MOFs were synthesized in one step via the hydrothermal method, exhibiting a fluorescence lifetime of 163 μs and spherical particles with diameters ranging from 250 to 400 nm. These conditions fulfill the characteristics and requirements of LFIA. Eu-MOFs exploit the porous nature of MOFs to mitigate the drawbacks associated with complex crosslinking agents. This enables antibody proteins to be cross-linked merely upon contact, thereby simplifying the detection process. A time-resolved LFIA method was developed utilizing Eu-MOFs for the detection of aflatoxin B1 (AFB1) in corn, achieving a limit of detection (LOD, IC10) of 0.149 ng/mL. The accuracy and reliability of the Eu-MOFs-LFIA method were validated through comparisons with spiked concentrations during spiking and blind sample analyses, with verification conducted using ultra-high-performance liquid chromatography mass spectrometry (UPLC-MS). Furthermore, testing of real samples demonstrated that the Eu-MOFs-LFIA method can effectively facilitate rapid detection of AFB1 in corn.

## 1. Introduction

Aflatoxin B1 (AFB1) is a type of heterocyclic aromatic secondary metabolite predominantly produced by *Aspergillus flavus* and *Aspergillus parasiticus* under humid and warm conditions [1]. It is estimated that 30–100% of corn is contaminated by AFB1 [2]. AFB1 is recognized for its highly carcinogenic, mutagenic, and teratogenic properties, as it can induce primary liver cancer, gastric cancer, and lung cancer [3,4,5]. AFB1 has been classified as a Group 1 carcinogen by the World Health Organization [6]. Consequently, numerous countries have established maximum residue limits (MRLs) for AFB1. China and the United States set the MRL for AFB1 in corn at 20 μg/kg [7]. Given the significant hazards posed by AFB1, there has been considerable attention to the rapid and highly sensitive detection of AFB1. Traditional detection methods for mycotoxins include thin-layer chromatography [8,9], high-performance liquid chromatography–tandem mass spectrometry [10,11], and ELISA [12,13]. However, these methods require skilled personnel, complicated sample pretreatment, and expensive instruments. Nevertheless, point-of-care detection of AFB1 is highly necessary, as it can significantly reduce detection costs and enable broader applications, particularly in food safety testing in remote areas. In scenarios with a high risk of AFB1 contamination, such as farmers’ markets, granaries, and edible oil storage stations, rapid detection can effectively mitigate the risk of AFB1 circulation. Therefore, developing a rapid, accurate, and highly sensitive method for AFB1 detection is essential.

Lateral flow immunoassay (LFIA) has been widely utilized across various fields due to its cost-effectiveness, ease of operation, and high specificity. Applications include early pregnancy testing, in vitro diagnostics, and the detection of pathogenic microorganisms. Recently, LFIA has attracted increasing attention in the domain of food safety [14]. During LFIA development, the use of nanoprobes as labeling materials has emerged as a significant area of research. Currently, the nanomaterials employed in LFIA encompass gold nanoparticles [15], quantum dots [16], carbon nanomaterials [17], and fluorescent microspheres [18]. However, these materials exhibit certain limitations in LFIA applications, including complex synthesis processes, high biotoxicity, and time-consuming protein labeling steps. Therefore, the development of fluorescent probes characterized by simple synthesis methods, ease of labeling, and time-resolved capabilities is essential for enhancing LFIA performance.

Metal–organic frameworks (MOFs) have been extensively applied in LFIA, with a predominant focus on visual detection methods [19,20]. While colorimetric and grayscale detectors have been employed for the quantitative analysis of LFIA, these techniques suffer from inadequate sensitivity. This limitation restricts their use in food safety inspections. Eu-MOFs have bloomed in the sensing area in the last decades because they possess highly stable and adjustable luminescence emission signals, high quantum yield, and long lifetime [21]. Fang Zhu developed a ratiometric fluorescence sensor by coordinating Eu^3+^ ions with ellipsoid-like Zr-LMOFs, achieving a significantly lower detection limit of 2.82 nM for AFB1 [22]. Even more encouraging is the advancement in portable fluorescence detection devices, which has significantly promoted the development of rapid fluorescence detection technology. In particular, the integration of fluorescence sensors with the inherent camera and image processing technology of smartphones enables more accurate on-site food safety analysis [23]. By utilizing 3D-printed casings, portable fluorescence detection systems that incorporate multispectral sensors, CMOS sensors, LEDs, and Raspberry Pi have been effectively applied for mycotoxin detection [24]. The application of Eu-MOFs in LFIA for detecting AFB1 in agricultural products represents a promising detection method.

**Figure 1:**
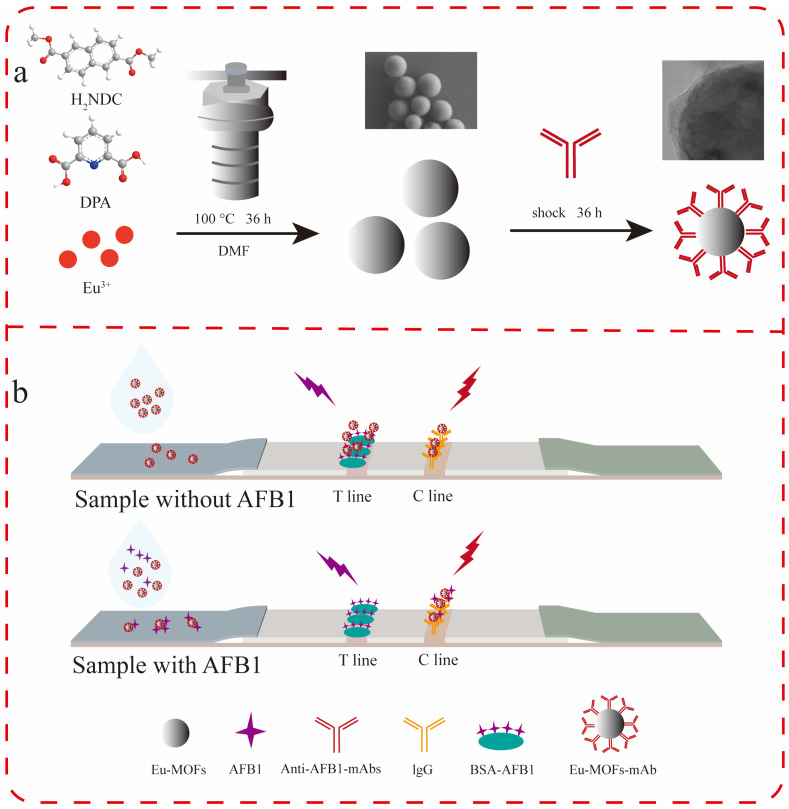
(**a**) Synthesis of Eu-MOFs. (**b**) LFIA assay for AFB1.

In this study, spherical Eu-MOFs with a uniform diameter were synthesized. MOFs exhibit excellent optical properties, a prolonged fluorescence lifetime, and strong stability in aqueous environments. These properties make them suitable probes for LFIA, as shown in Figure 1. Furthermore, they demonstrate favorable adsorption characteristics for proteins, allowing for straightforward antibody linkage through mere contact. This method presents a significant advantage over the dehydration condensation cross-linking mechanism typically associated with amide bonds, as it circumvents the complex linking process. In this study, Eu-MOFs were successfully utilized as a fluorescent label in the development of an LFIA method for the detection of AFB1 in corn, achieving a sensitivity of 0.149 ng/mL.

## 2. Materials and Methods

### 2.1. Materials and Instruments

The hexahydrate of europium nitrate, 2,6-pyridine dicarboxylic acid, and dimethyl 2,6-naphthalenedicarboxylate were obtained from Aladdin Biochemical Technology Co., Ltd. (Shanghai, China). Bovine serum albumin (BSA), aflatoxin B1 (AFB1), and AFB1 monoclonal antibodies were sourced from Shanghai Bio-Technology Co., Ltd. (Shanghai, China). T-2 toxin, ochratoxin A (OTA), deoxynivalenol (DON), and zearalenone (ZEN) were acquired from Meizheng Biotechnology Co., Ltd. (Rizhao, China). The secondary antibodies were supplied by Baosen Biotechnology Co., Ltd. (Beijing, China). 

All other reagents used were of analytical grade. Ultrapure water (18.25 MΩ) was used throughout the experiments. The CN 120 nitrocellulose (NC) membrane was procured from Millipore. Absorbent papers, coupling pads, sample pads, polyvinyl chloride (PVC) substrates, gold-plated film cutters, strip cutters, and immunochromatographic quantitative analysis instruments were sourced from Shanghai Jinbiao Biotechnology Co., Ltd. (Shanghai, China).

### 2.2. Preparation of Eu-MOFs

Eu-MOFs were synthesized via a modified hydrothermal method adapted from a previously reported procedure [25]. In brief, 16.8 mg of Eu(NO_3_)_3_·6H_2_O, 10 mg of 2,6-pyridine dicarboxylic acid, and 48.8 mg of dimethyl 2,6-naphthalenedicarboxylate were added to 5 mL of DMF and 0.75 mL of deionized water. The mixture was subjected to ultrasonication to ensure complete dissolution, followed by magnetic stirring at room temperature for 30 min. Subsequently, it was transferred into a 25 mL Teflon-lined stainless steel reactor and heated in an electric oven at 100 °C for 36 h. After cooling the mixture to room temperature, it was washed three times with DMF and three times with methanol. Finally, the product was dried in an oven at 60 °C, yielding a solid white powder.

### 2.3. Characterization

The crystal structure was characterized using X-ray diffraction (XRD). The surface composition and oxidation states of the elements were analyzed by X-ray photoelectron spectroscopy (XPS). The morphology and structure of the material were examined by applying scanning electron microscopy (SEM) and transmission electron microscopy (TEM). Furthermore, the optical properties of the Eu-MOFs were investigated utilizing ultraviolet–visible spectroscopy and fluorescence spectroscopy.

### 2.4. Preparation of Eu-MOFs-Abs Probe

AFB1 antibody (Abs) at a concentration of 1 mg/mL was added to 0.5 mL of the Eu-MOFs aqueous suspension. The centrifuge tube containing this mixture was shaken at room temperature for 30 min. Subsequently, 0.1 mL of BSA (10%, w/v) was added, and the mixture was shaken for an additional 15 min to block binding sites in the Eu-MOFs. Afterward, the mixture was centrifuged at 15,000 rpm for 10 min at 25 °C to remove the supernatant and unbound proteins. The resulting precipitate was resuspended in 0.2 mL of deionized water and stored in a refrigerator at 4 °C for subsequent experiments.

### 2.5. Preparation of Immunochromatographic Test Strips

The construction of the LFIA test strip was based on reported literature with appropriate modifications [26]. Specifically, the sample pad was blocked using a morpholineethanesulfonic acid (MES) buffer at pH 6.5, containing 2% BSA, and then cut into strips of appropriate width. The strips were fully immersed in the MES buffer and subsequently dried overnight in an oven at 37 °C, after which they were stored at 4 °C for later use. The absorbent pad was utilized without any pretreatment. AFB1 antigen at a concentration of 1 mg/mL and goat anti-mouse IgG (secondary antibody) at a concentration of 0.5 mg/mL were added to the NC membrane at a rate of 1 μL/cm to form the T line and C line, respectively. The NC membrane was then dried at 37 °C for 2 h. Following this, the sample pad and absorbent pad were sequentially assembled, overlapping the NC membrane by 1–2 mm. Finally, the constructed test strips were cut into 4 mm wide strips and stored at 4 °C.

### 2.6. Detection of Eu-MOFs-LFIA

The C line and T line were loaded with the secondary antibody and AFB1 antigen, respectively. The test sample and Eu-MOFs-Abs solution were added to the wells of the enzyme-linked immunosorbent assay (ELISA) plate and allowed to react for 2 min to facilitate the complete binding of AFB1 from the sample to the Eu-MOFs-Ab. Subsequently, the strip was inserted, and the test sample, along with the Eu-MOFs-Ab, migrated towards the NC membrane through chromatographic action. The Eu-MOFs-Ab probes bound to the AFB1 in the sample were not captured by the AFB1 antigen on the T line. Therefore, when the concentration of AFB1 in the test sample was sufficiently high, no visible fluorescence was observed on the T line. In contrast, when the AFB1 concentration was lower, the fluorescence intensity on the T line increased. For negative samples, the Eu-MOFs-Ab was predominantly captured at the T line by the AFB1 antigen, resulting in the strongest fluorescence observed. The fluorescent bands were visually detectable under a UV lamp for qualitative analysis. Quantitative analysis was performed using a fluorescent strip reader, with a detection delay time set to 10 μs.

The standard curve for Eu-MOFs-LFIA was established. A defined volume of Eu-MOFs-Abs probe was added to 100 μL of various concentrations of AFB1 standard solutions (0.01, 0.05, 0.1, 0.5, 1.0, 5.0, 10.0, and 50.0 ng/mL). These standard solutions were prepared using a corn matrix solution. The mixtures were allowed to react in ELISA wells for 2 min. Subsequently, the LFIA test strip was inserted into the mixed solution, and after a chromatographic reaction lasting 15 min, all probe liquids were absorbed onto the test strip through the chromatographic process. The fluorescence intensities of the T line and C line were measured using a fluorescence reader. Quantitative results were obtained through calculations to construct the standard curve, thereby determining the sensitivity of the Eu-MOFs-LFIA.

### 2.7. Specificity of Eu-MOFs-LFIA

This study focused on the verification of specificity in the detection of aflatoxin B1 (AFB1) and other mycotoxins detected by the Eu-MOFs-LFIA method. The mycotoxins selected for this investigation included aflatoxin B2 (AFB2), fumonisin B1 (FB1), zearalenone (ZEN), ochratoxin A (OTA), deoxynivalenol (DON), and T-2 toxin (T-2). Each reaction was conducted in triplicate, and the fluorescence intensities of the test line (T line) and control line (C line) were measured separately using a fluorescence reader. Subsequently, the FIT/FIC ratios were compared to evaluate specificity.

### 2.8. Verification Experiment

Corn was used as the actual sample. The test samples were analyzed using UPLC-MS to confirm the absence of AFB1 in the samples. The procedure for extracting the corn matrix was as follows: 10 g of the corn sample was ground and extracted with a methanol–water solution (8:2, v/v, 25 mL) using a vortex mixer for 10 min. A total of 5 mL of the extract was then concentrated using a rotary evaporator to dryness. The residue was reconstituted with a 10% methanol dilution solution and finally diluted 10-fold with deionized water for subsequent use.

## 3. Results and Discussion

### 3.1. Working Principle

To meet the requirements of LFIA fluorescent markers, the labeling materials should possess the following characteristics. The fluorescent material should have an appropriate particle size and porous structure, exhibit strong fluorescence excitation efficiency and excellent water dispersibility to prevent fluorescence quenching in aqueous environments, and have a prolonged fluorescence lifetime to enable time-resolved functionality. Due to the porous structure of MOFs, they can efficiently adsorb proteins, thereby improving labeling efficiency. The uniform characteristics of MOFs are appropriate to facilitate passage through the three-dimensional network pores of the NC membrane. Furthermore, some Eu-doped MOF materials exhibit instability in water and are prone to fluorescence quenching in aqueous solutions, highlighting the necessity for organic complexes that maintain good fluorescence in water [27]. According to characterization experiments, the synthesized Eu-MOFs meet the requirements for LFIA.

### 3.2. Characterization and Evaluation of Eu-MOFs

**Figure 2:**
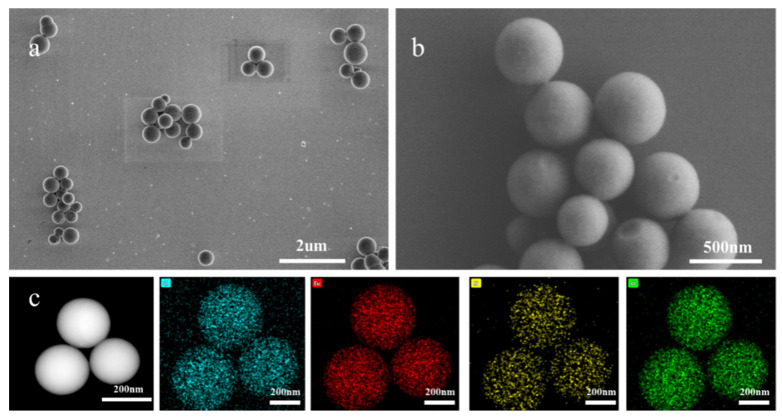
(**a**,**b**) SEM of Eu-MOFs under optimal conditions. (**c**) The energy spectrum from TEM.

The morphology of the synthesized Eu-MOFs was characterized using SEM and TEM. As illustrated in Figure 2 and Figure S1, the spherical diameter of the Eu-MOFs ranged from 250 to 400 nm, with an average particle size of 288.53 nm. The structure of the NC membrane comprises a three-dimensional fibrous network with pore sizes varying from 1 to 2 μm. If the size of the nanoprobe is excessively small, it flows too rapidly across the NC membrane, failing to achieve the desired chromatographic separation. Conversely, if the nanoprobe is excessively large, it is susceptible to clogging the NC membrane, which impairs the flow of the probe. Eu-MOFs exhibit a suitable size for optimal performance. Elemental mapping using TEM energy dispersive spectroscopy (EDS) was performed to investigate the elemental distribution within Eu-MOFs. The images reveal that primary elements, including carbon (C), oxygen (O), nitrogen (N), and europium (Eu), were uniformly distributed throughout the MOFs. An abundance of the Eu element was found in the MOFs, ensuring a high quantum yield for sensitive detection.

**Figure 3:**
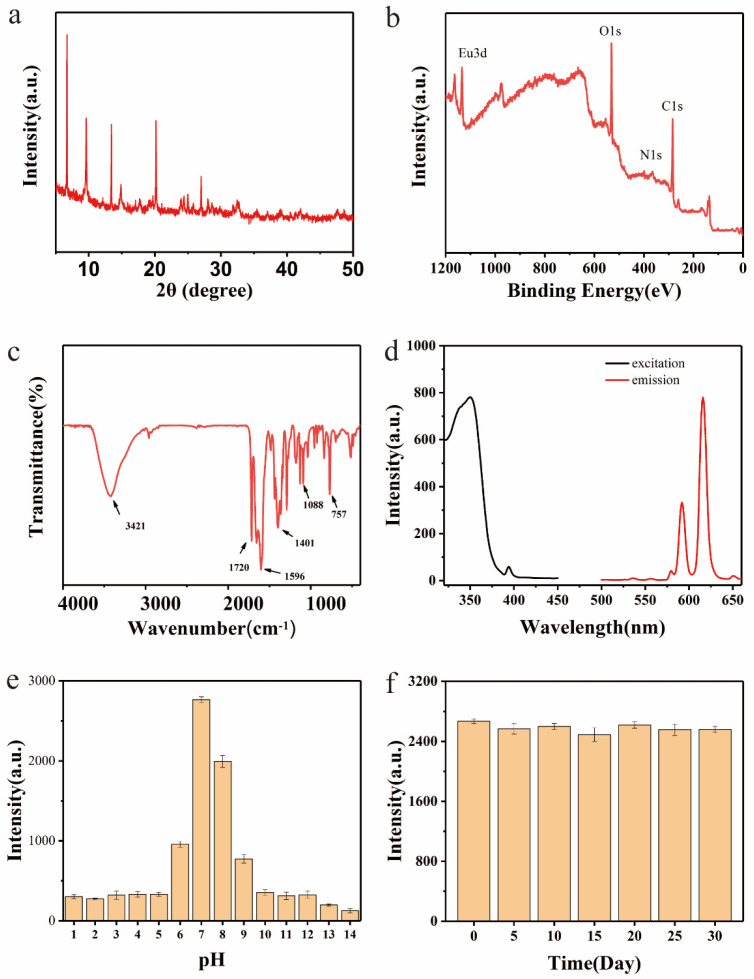
Eu-MOFs: (**a**) XRD; (**b**) XPS; (**c**) FT-IR; (**d**) excitation and emission spectra; (**e**) fluorescence intensity at different pH levels; (**f**) fluorescence intensity over different times.

The XRD pattern of the Eu-MOFs is presented in Figure 3a, revealing several characteristic peaks at 6.703°, 9.71°, 13.33°, 20.13°, and 27.02°. These peaks are comparable with the diffraction peaks observed in MOFs synthesized with 2,6-naphthalenedicarboxylic acid as the ligand [28]. The XPS analysis (Figure 3b) identified four distinct peaks at 532.19 eV, 399.32 eV, 284.80 eV, and 75.17 eV, which correspond to O1s, N1s, C1s, and Eu2p, respectively. The XRD results confirm the stable crystalline structure of Eu-MOFs, ensuring stability during detection.

In the FT-IR spectrum (Figure 3c), two Eu-O peaks were detected in the range of 1000–1100 cm^−1^, indicating the successful incorporation of Eu^3+^ into the MOF material. The region between 1400 and 1700 cm^−1^ displayed vibrational bands associated with carboxyl functional groups [29], with peaks at 1661 cm^−1^ and 1596 cm^−1^ corresponding to the asymmetric stretching of carboxyl groups [30]. Moreover, the presence of hydroxyl groups was evidenced by a prominent stretching vibration peak at 3421 cm^−1^. Additionally, due to the abundance of carboxyl groups in the Eu-MOFs, significant water dispersibility was exhibited. As shown in Figure S2 the fluorescence lifetime of Eu-MOFs measured by fluorescence spectra was 163 μs. The long fluorescence life of Eu-MOFs can be used for time-resolved analysis, which can effectively eliminate the background fluorescence of the environment and protein and improve the sensitivity of LFIA.

The fluorescence and excitation/emission spectra of the Eu-MOFs are presented in Figure 3d. The Eu-MOFs exhibited characteristic fluorescence emission peaks of Eu^3^⁺ at 615 nm and 590 nm. The excitation wavelength range was determined to be 325–370 nm, with the optimal excitation wavelength identified at 350 nm. Furthermore, to examine the impact of pH on the fluorescence stability of the Eu-MOFs, fluorescence intensity was measured across a pH range of 1–14. As depicted in Figure 3e, the Eu-MOFs demonstrate decreased stability in highly acidic or highly basic environments. The maximum fluorescence intensity was observed at a pH of 7.0. Interestingly, conditions near neutral pH are generally preferred to maintain antibody activity in LFIA. A pH of 7.0 is the optimal condition for Eu-MOFs-based LFIA. After storage at room temperature for one month, the fluorescence intensity of the Eu-MOFs exhibited no significant changes (Figure 3f), indicating that Eu-MOFs possess high stability, which will not negatively impact subsequent application development.

### 3.3. Eu-MOFs-Abs Labeling Evaluation

**Figure 4:**
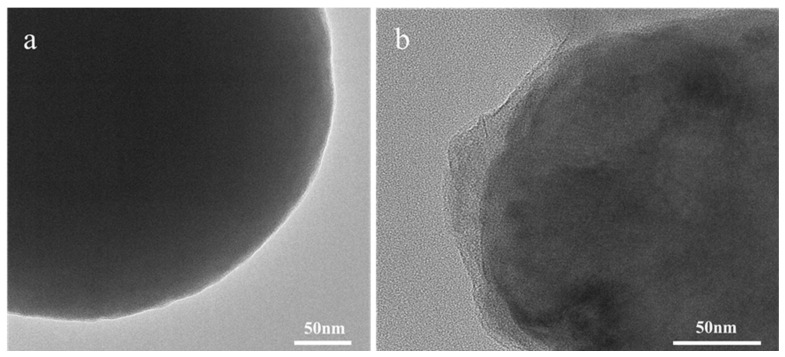
The TEM characterization of (**a**) Eu-MOFs and (**b**) Eu-MOFs cross-linked antibodies. The scale bar is 50 nm.

The antibodies were cross-linked to the Eu-MOFs through oscillatory contact, resulting in the preparation of Eu-MOFs-Ab probes, which significantly simplified the cross-linking process. As illustrated in Figure 4, the morphological changes of Eu-MOFs and Eu-MOFs-Abs were characterized using TEM. Figure 4a depicts the raw Eu-MOFs exhibiting a smooth edge. In contrast, Figure 4b displays the Eu-MOFs-Abs exhibiting a translucent protein layer distinctly enveloping the Eu-MOFs after antibody coupling. The Eu-MOFs is completely covered, with no smooth surfaces exposed, indicating that Eu-MOFs possess good biocompatibility and protein adhesion properties, which can help mitigate non-specific binding. Therefore, it can be concluded that the antibodies have been successfully coupled with the Eu-MOFs, rendering it suitable for subsequent experimental studies. Eu-MOFs contain positively charged metal ions, which enable them to bind with antibody proteins through electrostatic interactions and coordination [31]. The inherent porous structure and high surface area of MOFs are highly conducive to the immobilization and enrichment of biomolecules, thereby enhancing the stability of the binding [32].

### 3.4. Optimization of the Key Parameters of Eu-MOFs-LFIA

**Figure 5:**
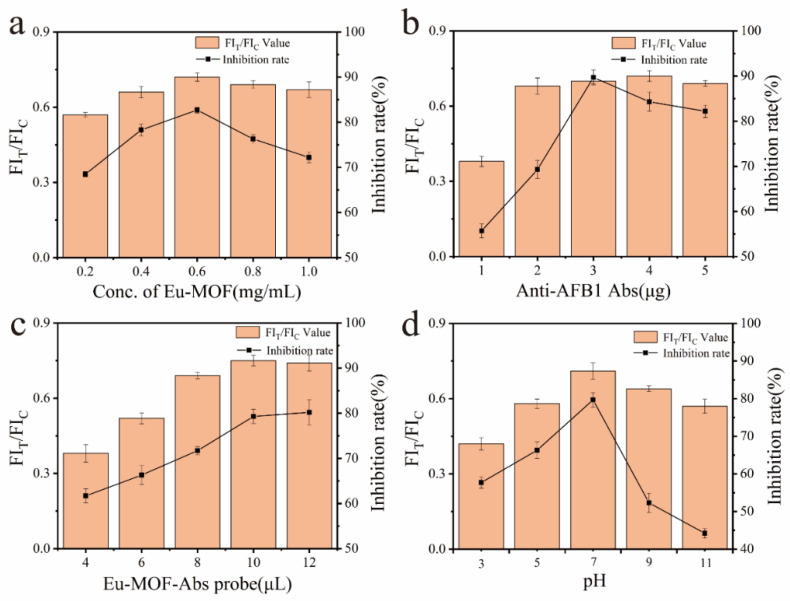
The effects of different conditions on FIT/FIC and inhibition rates: (**a**) Eu-MOFs concentration; (**b**) Eu-MOFs antibody content; (**c**) Eu-MOFs-Abs probe; (**d**) Eu-MOFs-Abs pH.

To achieve optimal performance of Eu-MOFs-LFIA, various parameters were systematically optimized, including the concentration of Eu-MOFs, the quantity of AFB1 antibodies, the volume of the Eu-MOFs-Ab probe, and the pH of the reaction. During the optimization process, all other conditions were maintained constant while a single variable was adjusted. Upon completion of the detection process using the test strips, the processed strips were placed in a fluorescence quantitative reader for the quantitative detection of AFB1. FIT and FIC denote the fluorescence intensities of the test line (T line) and control line (C line), respectively. The inhibition rate was calculated using the following formula: Inhibition Rate = (1 − B/B_0_) × 100%, where B_0_ and B represent the FIT/FIC values in the absence and presence of AFB1, respectively. As illustrated in Figure 5a, within the concentration range of 0.2–1.0 mg/mL, an increase in the concentration of Eu-MOFs correlated with a higher FIT/FIC value. However, the inhibition rate did not necessarily improve with higher concentrations of Eu-MOFs; it reached its maximum at a concentration of 0.6 mg/mL. Consequently, a concentration of 0.6 mg/mL is deemed optimal for Eu-MOFs.

Antibody concentration is a critical factor influencing LFIA performance. As illustrated in Figure 5b, the FIT/FIC and inhibition rates were analyzed for AFB1 antibody concentrations of 1, 2, 3, 4, and 5 μg. Notably, when the antibody concentration ranged from 1 to 4 μg, the FIT/FIC increased with increasing concentration. However, at a concentration of 5 μg, the FIT/FIC unexpectedly declined. This phenomenon may be attributed to the saturation of AFB1 binding sites on the T line. Once all AFB1 is bound by the antibody, no additional antibodies can attach, resulting in a maximum fluorescence intensity on the T line. Consequently, any unbound fluorescent probes are captured by the secondary antibody on the C line, which enhances the fluorescence intensity there and leads to a decrease in the FIT/FIC value. The highest inhibition rate was achieved at an antibody concentration of 3 μg, suggesting that this concentration is optimal.

The volume of the Eu-MOFs-Abs probe directly influences the fluorescence intensity at both the T and C lines of the test strip. The optimal working volume was determined by adjusting the quantity of the Eu-MOFs-Abs probe added, as illustrated in Figure 5c. When the volume was between 4 and 10 μL, the FIT/FIC gradually increased. However, a slight decrease in FIT/FIC was observed at a volume of 12 μL compared to 10 μL. The inhibition effect remained consistent, indicating that the Eu-MOFs probe volume reached a relatively balanced state at 10 μL, which can be confirmed as the optimal value.

The pH level serves as an indicator of the working environment for the probe and significantly influences both the fluorescence intensity of Eu-MOFs and the immunoaffinity of the antibodies. As illustrated in Figure 5d, both the FIT/FIC and inhibition rates were negatively impacted under acidic or basic conditions. Notably, when the pH was maintained at 7, the FIT/FIC and inhibition rates reached their peak values. Consequently, a pH of 7 was established as the optimal condition.

### 3.5. Analytical Performance of Eu-MOFs-LFIA

**Figure 6:**
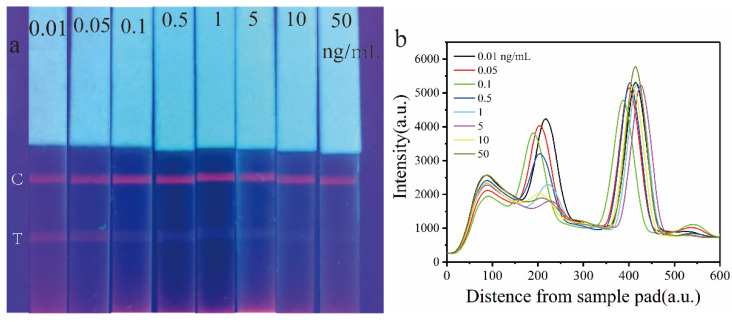
(**a**) The Eu-MOFs-LFIA test strip exhibits variations in fluorescence intensity at different concentrations of AFB1 (0.01, 0.05, 0.1, 0.5, 1, 5, 10, and 50 ng/mL) when subjected to ultraviolet light. (**b**) Spectrogram of the test strip obtained by a time-resolved fluorescence card reader.

Under optimized detection conditions, Eu-MOFs-LFIA was employed to detect AFB1 standard solutions in a corn matrix at spiked concentrations of 0.01, 0.05, 0.1, 0.5, 1, 5, 10, and 50 ng/mL. Figure 6a presents images of the visual test strips under UV light. As can be seen, the fluorescence intensity of the T line decreased with increasing AFB1 concentration, while the C line, which serves as the control line, consistently displayed fluorescence. The signal was calibrated using FIT/FIC. At an AFB1 concentration of 1 ng/mL, the fluorescence on the T line was notably weak; however, detection using a fluorescence reader revealed a measurable level of fluorescence intensity (Figure 6b). This finding indicates that the application of fluorescent probes offers a substantial enhancement in quantitative accuracy compared to traditional colorimetric methods.

**Figure 7:**
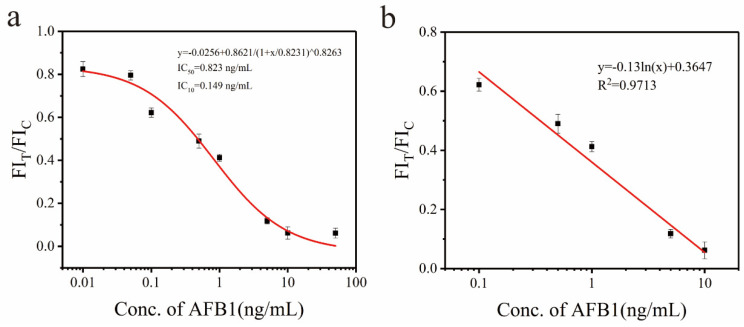
The quantitative curve of AFB1 detection in corn using Eu-MOFs-LFIA. (**a**) Corresponding relationship between the ratio of FIT/FIC and the AFB1 concentration. (**b**) The calibration curve for the Eu-MOFs-LFIA with the increasing concentration of AFB1 (from 0.1, 0.5, 1, 5, and 10 ng/mL) (*n* = 3).

Figure 7a illustrates the inhibition curve of the Eu-MOFs-LFIA, with FIT/FIC plotted on the vertical axis and AFB1 concentration on the horizontal axis. The points at which FIT/FIC exhibited a linear relationship with concentration were selected to construct the quantitative curve shown in Figure 7b. When the AFB1 concentration ranged from 0.1 to 10 ng/mL, the Eu-MOFs-LFIA demonstrated a strong linear correlation, characterized by the fitted linear equation *y* = −0.13*ln*(*x*) + 0.3647, *R*^2^ = 0.9713, where x and y are the concentration of AFB1 and the fluorescence ratio of the T lines and C lines. The limit of detection (LOD) was calculated as 0.149 ng/mL according to the IC10 value.

### 3.6. Specificity

Specificity is a critical criterion for evaluating the reliability of analytical methods. The specificity of Eu-MOFs-LFIA was validated by detecting various mycotoxins (AFB2, ZEN, DON, T-2, OTA, and FB1) alongside AFB1. AFB1 and other mycotoxins were added at a concentration of 100 ng/mL for the reaction. Subsequently, the completed test strips were placed into a fluorescence reader to measure and record the FIT/FIC values (*n* = 6). The results are illustrated in Figure S3. Under spiked conditions, AFB2, which possesses a molecular structure very similar to that of AFB1, exhibited comparable FIT/FIC values. In contrast, other mycotoxins were closer to the negative control. Therefore, the developed Eu-MOFs-LFIA method for detecting AFB1 in corn demonstrates high specificity.

### 3.7. Analysis of Real Samples by Eu-MOFs-LFIA and UPLC-MS

A total of 20 corn samples were randomly purchased from a local farmers’ market and analyzed using the developed Eu-MOFs-LFIA and UPLC-MS techniques. The Pearson correlation analysis method was employed to evaluate the detection results obtained from both methods. As illustrated in Figure S4, the results revealed a high correlation coefficient (*R*^2^ = 0.9557) between the detection values of the target analytes obtained through Eu-MOFs-LFIA and UPLC-MS. This indicates that the results of both methods were largely consistent in detecting AFB1 in corn samples, thereby confirming the reliability of the detection techniques used.

### 3.8. Actual Sample Testing

To evaluate the stability of the Eu-MOFs-LFIA method for AFB1 detection in corn, spiking recovery experiments were conducted. Various concentrations of AFB1 were added: 0.5, 5.0, and 10.0 ng/g. The recovery rates of the spiked samples ranged from 78.1% to 92.3%, with relative standard deviation (RSD) levels below 12.5% (Table S1). These results indicate that the established Eu-MOFs-LFIA method demonstrates good accuracy, meeting the national standard requirements for the maximum limit of AFB1 in corn. Compared to other rapid detection methods reported in the literature, the LOD) for AFB1 is lower than that of some previously published studies (Table 1).

## 4. Conclusions

Eu-MOFs were synthesized using the hydrothermal reaction method, and a Eu-MOFs-LFIA was successfully constructed for the detection of AFB1 in corn. Due to their advantages, including excellent biocompatibility, long fluorescence lifetime, stability in aqueous solutions with sustained strong fluorescence, and the ability to easily label antibodies through simple contact shaking, Eu-MOFs significantly enhance the fluorescence labeling efficiency of LFIA. The experimental results demonstrate that the Eu-MOFs-based LFIA method can effectively detect AFB1 in corn, with a detection limit as low as 0.149 ng/mL, achieving sensitivity at the level of the national standard for AFB1 monitoring. It is worth mentioning that the Eu-MOFs-LFIA detection process requires only 17 min in total and does not necessitate specialized skills. AFB1 in corn can be detected using a portable fluorescence card reader, rendering it suitable for widespread monitoring. This study provides a fluorescent labeling material suitable for LFIA, offering a new route to enhance the sensitivity and convenience of LFIA.

## Figures and Tables

**Table 1:** A comparison of the detection performance of our Eu-MOFs-LFIA method with previously reported LFIA methods in detecting mycotoxin.

Lable/Signal	Analyte	Linear Range (ng/mL)	LOD (ng/mL)	Real Samples	Reference
HPGN@4-MBA	AFB1	-	0.09	-	[33]
GNPs	FB1/ZEN/DON	-	60/6/12.5	Corn	[34]
Au@PDA	ZEN	-	0.0074	Corn	[35]
MGNH	OTA	0.098–12.5	0.094	Corn, wheat	[36]
Phycocyanin	AFB1	1–60	1.04	Wheat, corn, fodder	[37]
HfMOFs	T2	-	0.0487	-	[31]
PCFN	AFB1	0.2–48	0.16	Rice, bean, Peanut and lotus seed	[38]
Eu-MOFs	AFB1	-	0.149	Corn	This work

“-” represented “not found”.

## Data Availability

Data will be made available on request.

## Supplementary Materials

The following supporting information can be downloaded at https://www.mdpi.com/article/10.3390/bios15050313/s1: Figure S1: The particle size distribution map of Eu-MOFs; Figure S2: Fluorescence lifetime spectra of Eu-MOFs; Figure S3. The effect of different mycotoxins on the specificity of Eu-MOFs-LFIA; Figure S4. Correlation between Eu-MOFs-LFIA and UPLC-MSMS for detecting AFB1 in Corn; Table S1: Recovery rate of corn samples by adding standard experiment.

## References

[1] Song C., Yang J., Wang Y., Ding G., Guo L., Qin J. (2024). Mechanisms and transformed products of aflatoxin B1 degradation under multiple treatments: A review. Crit. Rev. Food Sci. Nutr..

[2] Zhang Y., Chen G., Chen X., Wei X., Shen X.-A., Jiang H., Li X., Xiong Y., Huang X. (2024). Aggregation-induced emission nanoparticles facilitating multicolor lateral flow immunoassay for rapid and simultaneous detection of aflatoxin B1 and zearalenone. Food Chem..

[3] Rushing B.R., Selim M.I. (2019). Aflatoxin B1: A review on metabolism, toxicity, occurrence in food, occupational exposure, and detoxification methods. Food Chem. Toxicol..

[4] Taranu I., Hermenean A., Bulgaru C., Pistol G.C., Ciceu A., Grosu I.A., Marin D.E. (2020). Diet containing grape seed meal by-product counteracts AFB1 toxicity in liver of pig after weaning. Ecotoxicol. Environ. Saf..

[5] Li H., Shang Q., Zhang L., Mao J., Zhang Q., Li P. (2024). Europium nanospheres based ultrasensitive fluorescence immunosensor for aflatoxin B1 determination in feed. Talanta.

[6] Wu Q., Xie L., Xu H. (2018). Determination of toxigenic fungi and aflatoxins in nuts and dried fruits using imaging and spectroscopic techniques. Food Chem..

[7] Wang Y., Wang X., Wang S., Fotina H., Wang Z. (2022). A Novel Lateral Flow Immunochromatographic Assay for Rapid and Simultaneous Detection of Aflatoxin B1 and Zearalenone in Food and Feed Samples Based on Highly Sensitive and Specific Monoclonal Antibodies. Toxins.

[8] Pestka J.J. (1991). High performance thin layer chromatography ELISAGRAM: Application of a multi-hapten immunoassay to analysis of the zearalenone and aflatoxin mycotoxin families. J. Immunol. Methods.

[9] Qu L.-L., Jia Q., Liu C., Wang W., Duan L., Yang G., Han C.-Q., Li H. (2018). Thin layer chromatography combined with surface-enhanced raman spectroscopy for rapid sensing aflatoxins. J. Chromatogr. A.

[10] Li J., Xu X., Guo W., Zhang Y., Feng X., Zhang F. (2022). Synthesis of a magnetic covalent organic framework as sorbents for solid-phase extraction of aflatoxins in food prior to quantification by liquid chromatography-mass spectrometry. Food Chem..

[11] Khayoon W.S., Saad B., Lee T.P., Salleh B. (2012). High performance liquid chromatographic determination of aflatoxins in chilli, peanut and rice using silica based monolithic column. Food Chem..

[12] Zhan S., Hu J., Li Y., Huang X., Xiong Y. (2021). Direct competitive ELISA enhanced by dynamic light scattering for the ultrasensitive detection of aflatoxin B1 in corn samples. Food Chem..

[13] Ye Y., Liu A., Wang X., Chen F. (2016). Spectra analysis of coating antigen: A possible explanation for difference in anti-AFB1 polyclonal antibody sensitivity. J. Mol. Struct..

[14] Liu S., Sun C., Zhang X., Shu R., Zhang J., Wang B., Wang K., Dou L., Huang L., Yang Q. (2025). Advances in enhancement-type signal tracers and analysis strategies driven Lateral flow immunoassay for guaranteeing the agri-food safety. Biosens. Bioelectron..

[15] Yang H., He Q., Lin M., Ji L., Zhang L., Xiao H., Li S., Li Q., Cui X., Zhao S. (2022). Multifunctional Au@Pt@Ag NPs with color-photothermal-Raman properties for multimodal lateral flow immunoassay. J. Hazard. Mater..

[16] Fang B., Xiong Q., Duan H., Xiong Y., Lai W. (2022). Tailored quantum dots for enhancing sensing performance of lateral flow immunoassay. TrAC–Trends Anal. Chem..

[17] Guo J., Chen S., Guo J., Ma X. (2021). Nanomaterial Labels in Lateral Flow Immunoassays for Point-of-Care-Testing. J. Mater. Sci. Technol..

[18] Lei X., Xu X., Wang L., Liu L., Kuang H., Xu L., Xu C. (2023). Fluorescent microsphere-based lateral-flow immunoassay for rapid and sensitive determination of eugenols. Food Chem..

[19] Li R., Bu T., Zhao Y.J., Sun X.Y., Wang Q.Z., Tian Y.M., Bai F.E., Wang L. (2020). Polydopamine coated zirconium metal-organic frameworks-based immunochromatographic assay for highly sensitive detection of deoxynivalenol. Anal. Chim. Acta.

[20] Xie W.Y., Tian M.L., Lun X., Jiang Y., He N., Liao X.L., Liu Y.S. (2020). A dual-mode fluorescent and colorimetric immunoassay based on in situ ascorbic acid-induced signal generation from metal-organic frameworks. Sens. Actuators B-Chem..

[21] Wang X., Jiang Y., Tissot A., Serre C. (2023). Luminescent sensing platforms based on lanthanide metal-organic frameworks: Current strategies and perspectives. Coord. Chem. Rev..

[22] Zhu F., Chai Q., Xiong D., Zhu N., Zhou J., Wu R., Zhang Z. (2024). Morphology Control of Zr-Based Luminescent Metal-Organic Frameworks for Aflatoxin B1 Detection. Biosensors.

[23] Fu W., Fu X., Li Z., Liu Z., Li X. (2024). Advances in smartphone assisted sensors for on-site detection of food safety based on fluorescence on-off-on mode: A review. Chem. Eng. J..

[24] Wang C., Gu C., Zhao X., Yu S., Zhang X., Xu F., Ding L., Huang X., Qian J. (2024). Self-designed portable dual-mode fluorescence device with custom python-based analysis software for rapid detection via dual-color FRET aptasensor with IoT capabilities. Food Chem..

[25] Wang Y., Zhang G.W., Zhang F., Chu T.S., Yang Y.Y. (2017). A novel lanthanide MOFs thin film: The highly performance self-calibrating luminescent sensor for detecting formaldehyde as an illegal preservative in aquatic product. Sens. Actuators B-Chem..

[26] Bao H., Yuan M., Xiao C., Liu D., Lai W. (2022). Development of a signal-enhanced LFIA based on tyramine-induced AuNPs aggregation for sensitive detection of danofloxacin. Food Chem..

[27] Mahata P., Mondal S.K., Singha D.K., Majee P. (2017). Luminescent rare-earth-based MOFs as optical sensors. Dalton Trans..

[28] Zheng X.J., Sun C.Y., Lu S.Z., Liao F.H., Gao S., Jin L.P. (2004). New porous lanthanide-organic frameworks: Synthesis, characterization, and properties of lanthanide 2,6-naphthalenedicarboxylates. Eur. J. Inorg. Chem..

[29] Yi K., Zhang L. (2021). Designed Eu(III)-functionalized nanoscale MOFs probe based on fluorescence resonance energy transfer for the reversible sensing of trace malachite green. Food Chem..

[30] Ma Y., Ni Y., Guo F., Xiang N. (2015). Flowerlike copper(II)-based coordination polymers particles: Rapid room-temperature fabrication, influencing factors, and transformation toward CuO microstructures with good catalytic activity for the reduction of 4-nitrophenol. Cryst. Growth Des..

[31] Zhang G., Liu T., Chen J., Yu S., Zheng Z., Deng S., Peng J., Lai W. (2025). Rigidifying aggregation-induced emission luminogens by metal–organic framework formation for sensitive lateral flow immunoassay. Talanta.

[32] Hu H., Wang Y. (2024). Recent advances in metal–organic frameworks as emerging platforms for immunoassays. TrAC–Trends Anal. Chem..

[33] Xie G., Liu L., Gong Y., Zhang G., Huang J., Xu H., Wang J. (2024). Development of tri-mode lateral flow immunoassay based on tailored porous gold nanoflower for sensitive detection of aflatoxin B1. Food Biosci..

[34] Hou S., Ma J., Cheng Y., Wang H., Sun J., Yan Y. (2020). One-step rapid detection of fumonisin B1, dexyonivalenol and zearalenone in grains. Food Control.

[35] Xu S., Zhang G., Fang B., Xiong Q., Duan H., Lai W. (2019). Lateral Flow Immunoassay Based on Polydopamine-Coated Gold Nanoparticles for the Sensitive Detection of Zearalenone in Maize. ACS Appl. Mater. Interfaces.

[36] Hao L., Chen J., Chen X., Ma T., Cai X., Duan H., Leng Y., Huang X., Xiong Y. (2021). A novel magneto-gold nanohybrid-enhanced lateral flow immunoassay for ultrasensitive and rapid detection of ochratoxin A in grape juice. Food Chem..

[37] Girmatsion M., Tang X., Zhang Q., Jiang J., Li P. (2024). Phycocyanin-based rapid fluorometric immunoassay for the determination of aflatoxin B1, deoxynivalenol, and zearalenone in food and feed matrices. Food Control.

[38] Li S., Zhong X., Xu Y., Zheng Y., Shi X., Li F., Guo S., Yang J. (2021). Smartphone-based reading system integrated with phycocyanin-enhanced latex nanospheres immunoassay for on-site determination of aflatoxin B1 in foodstuffs. Food Chem..

