# Supplementary material for: One-Step Labeling Based on Eu-MOFs to Develop Fluorescence Side-Flow Immunoassay for AFB1 Detection in Corn
Source: Biosensors (Basel). 2025 May 14;15(5):313. doi: 10.3390/bios15050313 (PMC12109949; doi:10.3390/bios15050313)
Supplement: Supplementary file 1 [file biosensors-15-00313-s001.zip › biosensors-3604335-supplementary.pdf]

biosensors

MDPI

Supplementary

One-Step Labeling based on Eu-MOFs to Develop Fluorescence Side-Flow Immunoassay for AFB1 Detection in Corn

Yinjun Li [1], Hua Ding [1], Ziyu Wang [2], Zewei Luo [2,*], Xitian Peng [1,*]

[1] Institute of Agricultural Quality Standards and Testing Technology Research, Hubei Academy of Agricultural Sciences, Hubei Key Laboratory of Nutritional Quality and Safety of Agro Products, Wuhan 430064, Hubei, PR China; yinjunli2015@163.com (Y.L.); dingh-15@163.com (H.D.)

[2] Research Center of Analytical Instrumentation, School of Mechanical Engineering, Sichuan University, Chengdu 610065; Sichuan, PR China; wangziyuscu@163.com

* Correspondence: zwluo@scu.edu.cn (Z.L.); pxitian@aliyun.com (X.P.)

Table of cintents

1. Supporting Figures

Figure S1. The particle size distribution map of Eu-MOFs.

Figure S2. Fluorescence lifetime spectra of Eu-MOFs.

Figure S3. The effect of different mycotoxins on the specificity of Eu-MOFs-LFIA.

Figure S4. Correlation between Eu-MOFs-LFIA and UPLC-MSMS for detecting AFB1 in Corn.

2. Supporting Tables

Table S1. Recovery rate of corn samples by adding standard experiment.

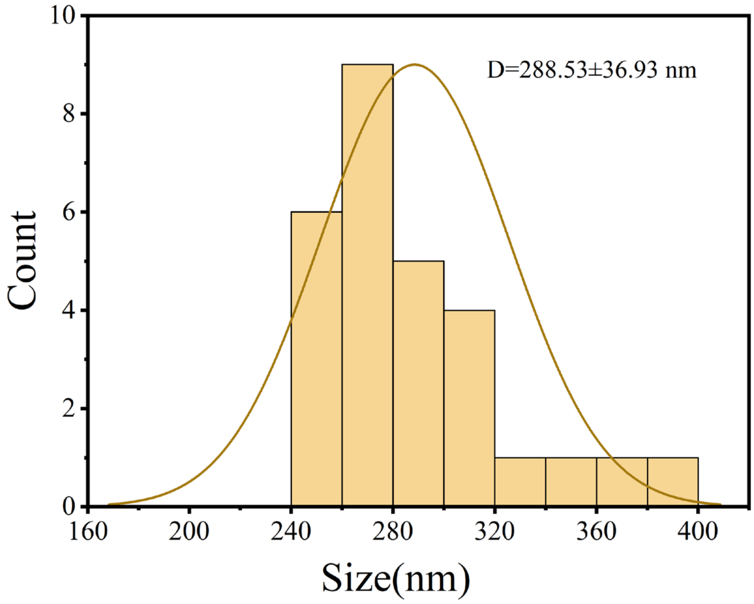

Figure S1. The particle size distribution map of Eu-MOFs.

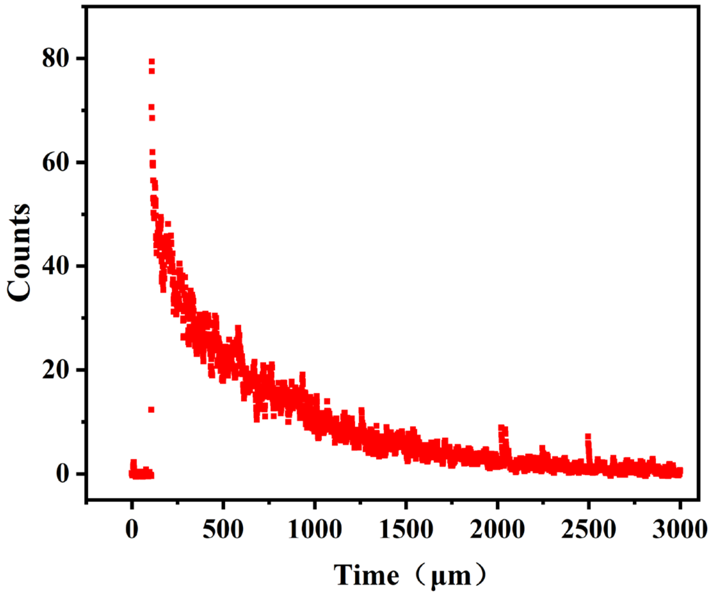

Figure S2. Fluorescence lifetime spectra of Eu-MOFs.

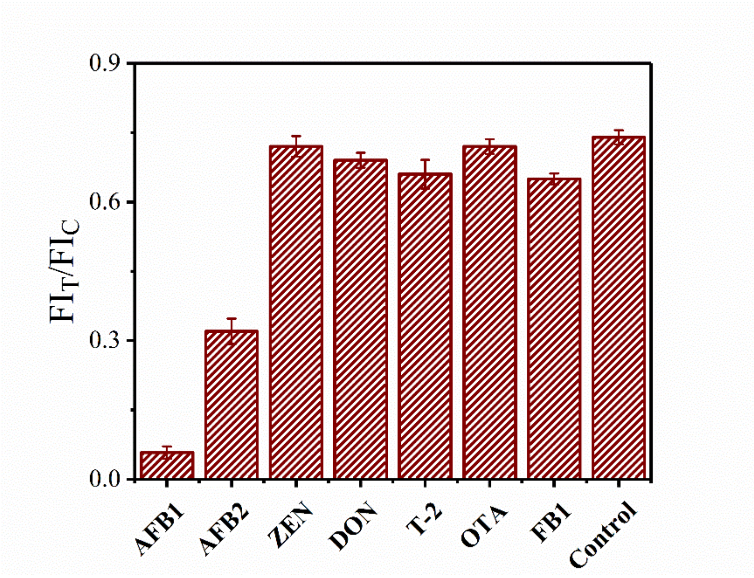

Figure S3. The effect of different mycotoxins on the specificity of Eu-MOFs-LFIA.

Table S1. Recovery rate of corn samples by adding standard experiment.

Sample	Spiked concentration (ng/g)	Recovery (%)	RSD (%)
	0.5	78.1	7.5
Core	5	92.3	8.6
	10	81.3	12.5

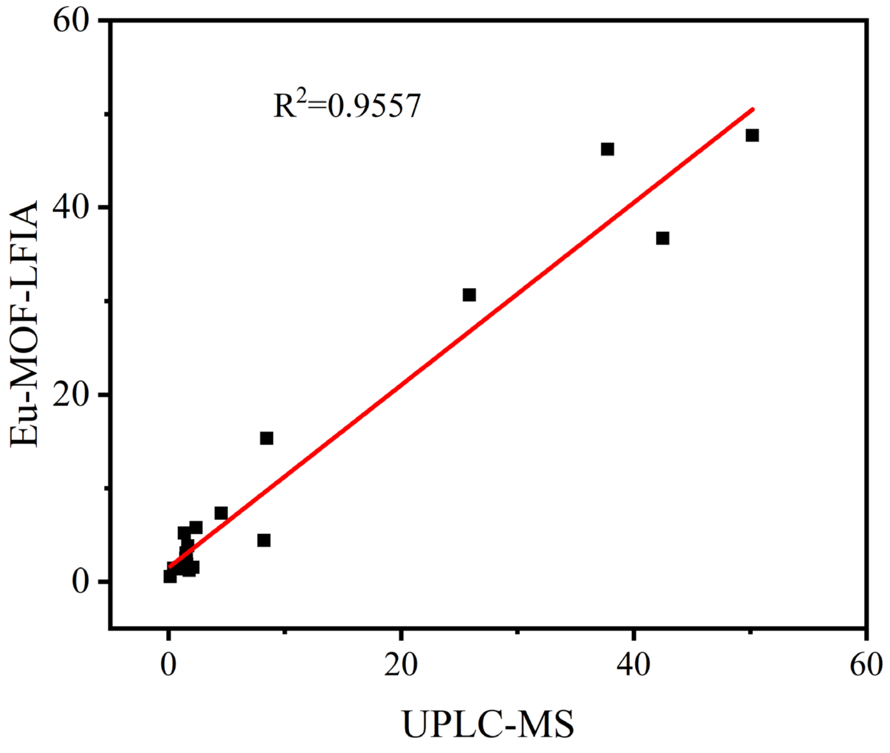

Figure S4. Correlation between Eu-MOFs-LFIA and UPLC-MSMS for detecting AFB1 in Corn.
